# Endoscopic endonasal pituitary surgery: How we do it. Consensus statement on behalf of the EANS skull base section

**DOI:** 10.1016/j.bas.2023.102687

**Published:** 2023-10-08

**Authors:** Ilaria Bove, Domenico Solari, Michaël Bruneau, Moncef Berhouma, Emmanuel Jouanneau, Jan Frederick Cornelius, Mahmoud Messerer, Roy Thomas Daniel, Sebastien Froelich, Diego Mazzatenta, Torstein Meling, Dimitrios Paraskevopoulos, Pierre-Hugues Roche, Henry W.S. Schoeder, Idoya Zazpe, Massimiliano Visocchi, Ekkehard Kasper, Marcos Tatagiba, Luigi Maria Cavallo

**Affiliations:** Department of Neurosurgery, University of Naples Federico II, Naples, Italy; Department of Neurosurgery, Universitair Ziekenhuis Brussel (UZ Brussel), Vrije Universiteit Brussel (VUB), Brussels, Belgium; Department of Neurosurgery, University Hospital, Dijon, Bourgogne, France; Neurosurgery Department, Hôpital Neurologique Pierre Wertheimer, Hospices Civils de Lyon, France; Department of Neurosurgery, Medical Faculty, Heinrich-Heine-University, Düsseldorf, Germany; Department of Neurosurgery, Department of Neuroscience, Centre Hospitalier Universitaire Vaudois, University Hospital, Lausanne, Switzerland; Department of Neurosurgery, Hôpital Lariboisière, Assistance Publique - Hôpitaux de Paris, Université Paris - Cité, Paris, France; Department of Neurosurgery, Neurological Sciences Institute IRCCS, Bologna, Italy; Department of Neurosurgery, The National Hospital, University of Copenhagen, Copenhagen, Denmark; Department of Neurosurgery, Barts Health NHS Trust, St. Bartholomew's and the Royal London Hospital, Blizard Institute QMUL, London, United Kingdom; Service de Neurochirurgie, Aix-Marseille Université, Assistance Publique-Hôpitaux de Marseille, Hôpital Nord, Marseille, France; Department of Neurosurgery, University Medicine, Greifswald, Germany; Department of Neurosurgery, University Hospital of Navarre, Pamplona, Spain; Department of Neurosurgery, Institute of Neurosurgery Catholic University of Rome, Italy; Department of Neurosurgery, St. Elizabeth Medical Center and Dana Farber Cancer Institute, Brighton, USA; Department of Neurosurgery, University Hospital Tübingen, Eberhard-Karls-University, Tübingen, Germany; Department of Neurosurgery, University of Naples Federico II, Naples, Italy

**Keywords:** Pituitary adenoma, Pituitary neuroendocrine tumor, Pituitary surgery, Endoscopic endonasal, Skull base, Survey, EANS

## Abstract

**Introduction and research question:**

The use of an endoscope in skull base surgery provides a panoramic close-up view over the intracranial structures from multiple angles with excellent illumination, thus permitting greater extent of resection of tumors arising at sellar area, mostly represented by PitNet - Pituitary neuroendocrine tumors, with higher likelihood of preserving vital/intact gland tissue. For this refined specialty of neurosurgery, unique skills need to be acquired along a steep learning curve.

**Material and methods:**

EANS (European Association of Neurosurgical Societies) skull base section panelists were enrolled and 11 completed the survey: the goal was to provide a consensus statement of the endoscopic endonasal approach for pituitary adenoma surgery.

**Results:**

The survey consisted of 44 questions covering demographics data (i.e., academic/non-academic center, case load, years of experience), surgical techniques (i.e., use of neuronavigation, preoperative imaging), and follow-up management.

**Discussion and conclusions:**

In this paper we identified a series of tips and tricks at different phases of an endoscopic endonasal pituitary surgery procedure to underline the crucial steps to perform successful surgery and reduce complications: we took in consideration the principles of the surgical technique, the knowledge of the anatomy and its variations, and finally the importance of adjoining specialties experts.

## Highlights

• Peer knowledge of anatomy of the approach is mandatory.
• The use of neuronavigation and/or micro-Doppler probe is highly recommended especially in case of conchal type of sphenoid sinus and recurrent cases.
• Adequate instruments maneuverability during the procedure should be considered: binostril or monostril approach are tailored upon need.
• Autologous materials, mucoperichondrium and naso-septal flap preferred according to the grade of intraoperative CSF leak.
• Postoperative endocrinological strict monitoring is suggested and naso-septal flap preferred according to the grade of intraoperative CSF leak.

## Background

The use of an endoscope in skull base surgery provides a panoramic close-up view over the intracranial structures from multiple angles with excellent illumination, thus permitting greater extent of resection of tumors arising at sellar area, mostly represented by PitNet - Pituitary neuroendocrine tumors, with higher likelihood of preserving vital/intact normal gland tissue. For this refined specialty of neurosurgery, unique skills need to be acquired along a steep learning curve.

In this paper we identified a series of tips and tricks at different phases of an endoscopic endonasal pituitary surgery procedure to underline the crucial steps to perform successful surgery and reduce complications: we took into consideration the principles of the surgical technique, the knowledge of anatomy and its variations and the importance of adjoining specialties experts.

## 1 Introduction

Pituitary adenomas (PAs) account for approximately 10–15% of all intracranial neoplasms and represent the third most frequent primary brain tumor in humans (Asa et al., 2017): they are a heterogenous group of tumors with complex clinical features and a wide range of secreting activities and possibly aggressive behavior. Diagnosis and classification of PAs have been historically based on their functional status and conventional histopathological staining of anterior pituitary hormones. “Functioning” PAs produce an excess of hormones leading to clinical signs, whilst “non-functioning” PAs don't produce measurable amounts of circulating intact hormones. Recently, the term pituitary neuroendocrine tumors (PitNETs) was proposed to replace the term pituitary adenoma to underline their unpredictable behavior (Asa et al., 2022) (Asa et al., 2020). Since the 2017 fourth edition of the WHO classification of CNS tumors, the classification of PitNET is based on immunohistochemical expression patterns of pituitary hormones and on the presence of pituitary-specific transcription factors (e.g., PIT-1, SF-1, TPIT) (Mete and Lopes, 2017).

Based on radiological appearance, PAs are classified as microadenomas (<10 mm in diameter, generally contained within the pituitary gland and sella, less frequently invasive), macroadenomas (≥10 mm in diameter, enclosed by sellar boundaries, expansile, or invasive) or giant (≥40 mm in diameter, dumbbell-shaped, multilobulated, or with asymmetrical extension beyond the sellar boundaries) (Cossu et al., 2022).These tumors can be diagnosed due to hormonal abnormalities such as hypersecretion or deficiencies and/or per the presence of clinical signs related to mass effect or even discovered incidentally. Despite no histological differences have been ever detected, invasive tumors grow faster, leading to eventual infiltration of neighboring structures such as the dura mater, bone, sphenoid and surrounding areas of the skull base (Ng et al., 2021). Sometimes it is difficult to assess the degree of tumor invasion, as PAs do not present a true capsule rather a “pseudo-capsule” formed by pituitary lining cells and the reticulin network that is not recognizable when tumor breaches out of the sella. Surgical removal is the treatment of choice in most of the cases. In Prolactinomas, surgery is considered appropriate in cases of dopamine-agonist drugs (DA)resistance, DA intolerance, spontaneous or DA-induced CSF leakage, or for patients who are unwilling to undergo chronic medical treatment (Cozzi et al., 2023)

Sometimes complete tumor resection is impossible to accomplish; many cases require a multimodal treatment strategy which includes pharmaceutical support, and eventual adjuvant fractionated stereotactic radiotherapy (SRT) or stereotactic radiosurgery (SRS), to achieve adequate long-term disease control.

### 1.2 Materials and methods

EANS skull base section panelists were enrolled and 11 completed the survey: the goal was to provide a consensus statement of the endoscopic endonasal approach for pituitary adenoma surgery. The survey consisted of 44 questions covering demographics data (i.e., academic/non-academic center, case load, years of experience), surgical techniques (i.e., use of neuronavigation, preoperative imaging), and follow-up management.

### 1.3 Goals of surgery in pituitary adenomas

The preoperative diagnosis, the management, and treatment of pituitary adenomas candidates for surgery require a multidisciplinary approach involving a team of endocrinologists, neurosurgeons, otolaryngologists, neuro-ophthalmologists, endocrine pathologists, neuroradiologists, and radiotherapists with expertise in pituitary disease. The multidisciplinary approach optimizes preoperative and hormonal follow-up, ophthalmological, and radiological evaluation; improves surgical outcomes, minimizing complications and facilitating the proper adjuvant treatment. Upon neurological and endocrinological symptoms, the main goals of surgical treatment for patients with adenomas should be (Asa et al., 2017; Cappabianca et al., 2014a; Ebersold et al., 1986; Laws and Thapar, 1999; Wilson, 1990):

- A maximal safe tumor removal, to grant the relief of mass effect and/or hormonal hypersecretion signs;
- The restoration or preservation of physiological neurological functions particularly visual functions;
- The decompression of the pituitary gland to improve or preserve remaining hormonal functions.

Pituitary surgery has progressed through a long journey with the evolution of transsphenoidal route from a predominantly microscopic to mostly endoscopic approach (Cappabianca et al., 2004; Messerer et al., 2011). Technical advances and the development of new technologies have favored better surgical outcomes along with fewer complications, and several advantages for the patients and the surgeons as reported in pertinent literature over the past two decades (Zada et al., 2011; Kassam et al., 2005a, 2005b; Aydin et al., 2007; Cappabianca et al., 2008). The dynamic visualization achieved by the endoscope, from a wide panoramic to a close-up view, and the capability to “look around corners”, has further expanded the surgical horizons of this technique.

## 2 Results

The first group of questions was regarding the experience with pituitary surgery. Concerning the caseload per center/year (Q4 “Endoscopic pituitary surgery case load/year”) 63.6% of the survey participants performed >100 surgeries per year, 18.2% 50–100 surgeries, and 18.2% < 50 surgeries per year, with 63.6% reporting experience with pituitary surgery “>20 years” and 36.4% “15–20 years”, (Q1 “How many years of pituitary surgery experience?“). These data reflect the heterogeneity of experience among different surgeons, despite this, a common current practice of pituitary surgery among neurosurgical departments in Europe is reported.

**Fig. 1:**
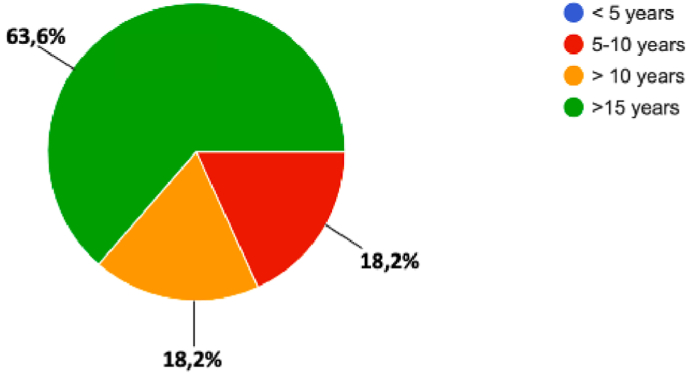
Question 3 “When did you start endoscopic pituitary surgery?”

The endoscope was adopted in 100% of the participants: in 63.6% of respondents “>15 years”, 18.2% “5–10 years”, and 18.2% “>10 years” (Q3 “When did you start endoscopic pituitary surgery?“) [Fig. 1]; 63.6% had performed previous microscopic procedures, whilst 36.4% did not (Q2 “Previous experience with microscopic pituitary surgery”).

## 3 Preoperative planning

### 3.1 Understanding anatomy

Preoperative MRI is necessary to obtain details of the anatomical configuration and size of the nasal airway (i.e. deviated nasal septum, concha bullosa) and the paranasal sinuses (i.e. sphenoid sinus pneumatization or variations such as sellar, presellar or conchal types, intra-/inter-sphenoid septa) and sella (anatomical variations, kissing carotids or bone dehiscence). For the majority of respondents, the sphenoid sinus anatomy (Q6 Sphenoid sinus anatomy is a limit? When) is not a thorough contraindication for the endoscopic endonasal approach (63.6%), whilst 27.3% and 9.1% of them defined respectively a choncal and a presellar sinus type a contraindication. None of those who answered, consider a narrow intracavernous intercarotid distance (Q7 A narrow intracavernous intercarotid distance – if yes, in which cases?) a contraindication for the approach. Computed tomography (CT) scans might be useful in studying this bony anatomy.

The analysis of peculiar MRI features can provide hints in regard to the consistency of the tumor (which is likely to be firm if it is hypointense on T2 images), the pituitary gland and stalk position and the CS degree of involvement. Regarding this last point, PITNETs can be categorized into five groups according to Knosp classification (Knosp et al., 1991): the higher is the grade, the lower are the chances of gross total resection (GTR). Hence, (Q5 “Which preoperative imaging do you usually perform?“) most respondents (90,9%) perform “Both (MRI + CT scan)”, at preoperative stage. Encasement of vascular structures and cavernous sinus invasion do not represent a contraindication to the approach, but it is recommended to implement radiology with angiographic sequences (MRA, Angio-CT, DSA) to detail relationships, especially in redo surgeries.

### 3.2 Neuronavigation and Doppler probe

**Fig. 2:**
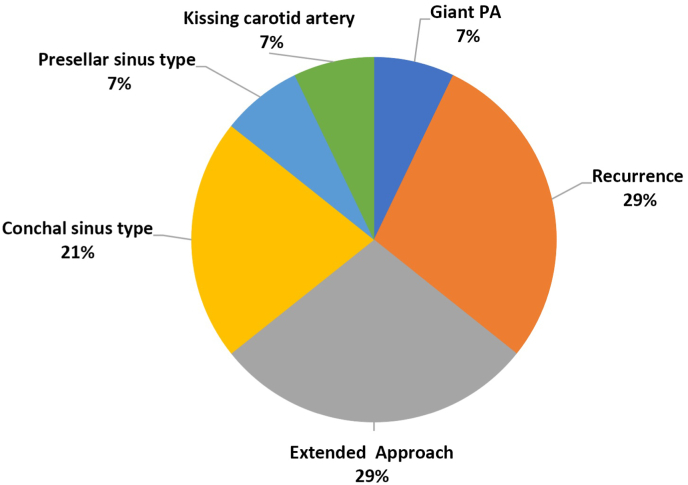
Question 8 “In which cases do you use neuronavigation?”

The use of neuronavigation is mostly recommended in peculiar cases such as anatomical variants that alter the corridor or in cases of tumor recurrence or in the presence of large/invasive lesions involving the suprasellar and/or parasellar regions. Accordingly, most of answers (72.7%) concerning the use of navigation (Q8 “Are you using a neuronavigation systematically?” – percentage of cases and why) - confirmed that it is required/used upon selected cases, most common of which are disclosed in Fig. 2.

**Fig. 3:**
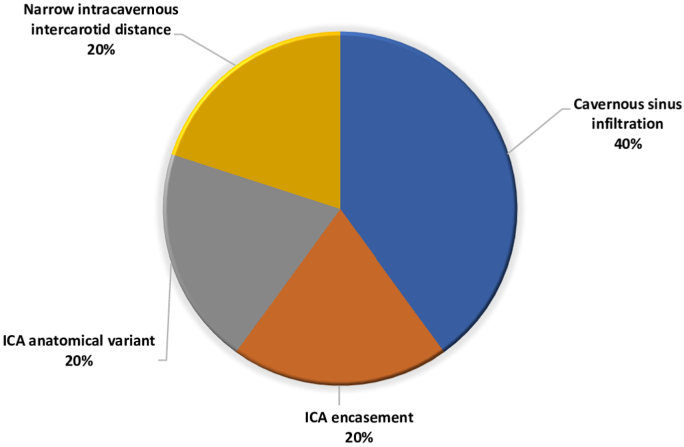
Question 9 “In which cases do you use micro doppler probe?”

The use of micro-doppler probe can be useful to depict the course of ICA, especially in case of parasellar extension of the tumor, or in children, the use of a doppler probe is advised due to the significantly narrow intercarotid corridor as compared to adults (Banu et al., 2014; Rastatter et al., 2015; Tatreau et al., 2010), albeit lack of significant differences has been reported starting at the age of 9 (Tatreau et al., 2010). Hence, 90.9% of the respondents use it (Q9 “Are you using a micro doppler probe?” – if yes in which cases?), usually in case of cavernous sinus infiltration, encasement of ICA or its anatomically variant, and/or narrow intracarotid distance, as shown in Fig. 3.

### 3.3 Preoperative considerations

The extended endoscopic endonasal approach differs from the standard since the first phases: it requires a creation of a wider surgical corridor by mean of unilateral middle turbinate resection, posterior bilateral ethmoidectomy, and posterior septectomy. Watertight and resilient repair of the skull base osteo-dural breach should be taken into account. Concerning the use of a preoperative lumbar drain to prevent a CSF leak (Q10 “Do you use lumbar drain?“), 63.6% of respondents prefer not to; those who adopt it reported circumstances and reasons as follow: high flow CSF leak during surgery, revision surgery, and in case of major CSF leak during surgery when mucosal flap is not available. For reconstruction purpose, fat of fascia lata can be harvested so at this time a small periumbilical or thigh region are prepped.

### 3.4 Operating room set-up and Surgeon's position

The OR set up is also a crucial aspect to make surgery efficient: the endoscopic equipment is placed ergonomically to be at hand for best convenience of the surgeons and all staff; the anesthesiologist is usually positioned with his/her equipment at the opposite side of the patient at the level of the head, but he/she can also be positioned at the patient's feet.

The optimal surgical setting of an EEA for safe maximal resection is a matter of discussion, and the environmental conceptualization for endoscopic endonasal skull base procedures is based according to surgeon's preference and experience.

If the surgeon is right-handed, he/she will prefer to use the patient's left nostril for his/her crucial surgical instrument maneuvers and the endoscope will be allotted in the right nostril; similarly, if the surgeon is left-handed, he/she will prefer to use the patient's right nostril for his/her the use of crucial surgical instruments and the other surgeon can be more comfortably placed on the other side. At the initial phase of the procedure, the endoscope is held in the nondominant hand, and main instrument, including the dissectors, rongeurs, and shavers in the dominant hand. From the sphenoid phase over, above all during tumor dissection and removal maneuvers, the 4-hands/2-nostrils technique represent the ultimate policy: the endoscope is held by the coworker in a nostril that is stretched upward (at 12 o'clock) with another instrument (usually a suction – held by first surgeon) in the most inferior position in the same nostril (at 6 o'clock). In this scenario respondents resulted mostly adherent to this concept preferring either to work “with an ENT colleague”, or “with an ENT or “neurosurgeon resident” or “alone” each apart in 27.3% of cases, and in 18.2% the team is composed by “Two neurosurgeons” (Q11 “Who do you usually perform the procedure with”).

**Fig. 4:**
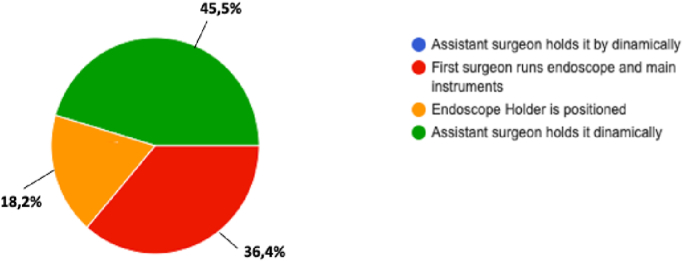
Question 15 “From the sphenoid phase over, how do you deal with the endoscope”.

Contrariwise, after completing the nasal phase there is a percentage of surgeons who might prefer to continue surgery with the aid of fixed holder for endoscope: indeed, 45.5% performed the maneuvers with the aid of assistant, in 36.4% first surgeon runs endoscope and main instrument, and in 18.2% the endoscope holder is positioned (Q15 “From the sphenoid phase over, how do you deal with the endoscope”) [Fig. 4].

The surgeon holding the endoscope moves in and out and rotates it if necessary, as to constantly adjusts position according to the surgical target and increase maneuverability of the instruments of the first surgeon. However, conflicts between the endoscope and the instruments can occur and are even more pronounced when the endoscope is close to the tip of the instruments: this can lead to so-called “rolling” between the two instruments by slipping one on top of the other. The team needs to be perfect tuned in dynamics and synchronicity to reduce the “conflict of sword”, so proper coordination is a thoughtful skill to be trained along.

### 3.5 Patient's positioning

The patient is positioned (Q12 “surgical positioning”) supine with the trunk raised to about 10°, the head it is slightly turned towards the surgeon, as per most common attitude (45.5%); the head is in neutral position in case of a standard approach, or extended 10–20° in cases of anterior skull base approaches, or flexed 10–20° for reaching a lower target as clival and cranio-vertebral junction area. Mayfield-type fixation is adopted whether optical image-guided system is used, but not in case of electromagnetic (EM) neuronavigation. Routine disinfection of the nasal area is performed and cottonoids soaked with a decongestant/anesthetic solution whose ratio and components might vary (Q13 “What do you use for mucosal vasoconstriction?“) (e,g., 1 mg of adrenaline, 5 ml of 20% diluted lidocaine and 4 ml of saline solution) are inserted in both nostrils.

Nasal preparation may vary from different center, for example in the United Kingdom (UK) the Moffet's solution (Cocaine Hydrochloride 10 per cent 2 ml, Sodium Bicarbonate 1 per cent 2 ml, 1:1000 Adrenaline ml) remains still available; the combined vasoconstrictor and decongestant effects of the symbiotic cocaine and adrenaline together with the synergistic effect of sodium bicarbonate provide an excellent surgical field for rhinologic procedures (Benjamin et al., 2004).

### 3.6 Endoscope and equipment

Surgery is performed under direct endoscopic vision, employing a 0°, 18 cm long, 4 mm of diameter (2.7 mm in pediatric cases) scope; 30° and/or 45° endoscopes are also used to visualize most lateral aspects of lesion and work in the hidden corners, as at the end of surgery for the inspection of the tumor cavity. For this reason, straight instruments (not bayonet shaped) slightly curved at the tip (e.g., suction device and bipolar cautery) are preferred. Use of an external irrigation sheath of the endoscope permits irrigation and continuous cleansing of the lens, avoiding the “in and out” movement through the nasal cavity. This is the most preferred method (Q14 “What do you use for lens cleaning?“), as per survey results (72.7%), whilst 18.2% use “External irrigation” and only 9.1% “Wipe outside” the lens.

## 4 Surgical procedure

### 4.1 Initial steps: creation of the corridor and adequate exposure

The nasal phase is fundamental for the next steps as several issues during the tumor removal can be related to inadequate exposure: the nasal phase starts with the insertion of the 0°or 30° endoscope into the right nostril to identify the main anatomical landmarks: the inferior turbinate, the septum, and the middle turbinate. The endoscope is moved forward to have into the view the roof of the choana and the spheno-ethmoid recess, where the sphenoid ostium is identified. Once the choana has been identified, entry into the sphenoid sinus can essentially take place through two routes: through the rostrum of the sphenoid sinus or through the natural ostium of the sphenoid sinus. The mucosa that covers the anterior wall of the sphenoid sinus is dissected, taking care of preserving the septal branches of the sphenopalatine artery, which is essential for a healthy naso-septal flap. Sphenoidotomy is performed ca 2.5 mm above the roof of the choana up to the sphenoid ostium and along the posterior portion of the nasal septum. In these regards, 54.5% of respondents (Q17 “How do you enter into the sphenoid sinus?“) favor the “Enlarging the natural ostium of the sphenoid sinus” to enter sphenoid sinus, while the 45.5% prefer “Drilling at rostrum of the sphenoid sinus”.

Thereafter, the nasal septum is detached from the sphenoid bone; a wide sphenoidotomy is performed, along with complete removal of the rostrum and flattening of the floor of the sphenoid sinus. In case of two nostril approach, posterior portion of nasal septum is removed to create unique binasial corridor. Mononostril approach without nasal septum removal can limit nasal discomfort, but on the other side the binostril approach offers a wider exposure over the anatomy of the skull base and eventually a superior maneuverability of two instruments. Extensive manipulation of the nasal septum and the ethmoid is not recommended as per the risk of damaging the olfactory nerve fibers or ventilatory complications leading to severe postoperative discomfort. For these reasons, no strategy (Q16 “What kind of technique do you mainly use”) is univocal addressed as superior with slight preference for monostril technique (54.5%) rather than the binostril technique (45.5%).

### 4.2 Instruments maneuverability inside the sphenoid sinus

The opening of the sphenoid sinus must be wide enough to allow adequate exposure but above all to grant safe maneuverability of surgical instruments at surgical target. In the presence of favorable conditions (i.e., wide nasal corridor), working through one nostril is possible. The lateral limit of a wide sphenoidotomy is the lateral wall of sphenoid sinus, and the superior limit is the planum sphenoidale. Inferiorly, the dissection is continued to provide adequate working space below the sellar floor (clival recess). To obtain adequate orientation and prepare the sphenoid for drilling, the mucosa inside the sphenoid sinus can be completely removed or just along the breach. This latter solution it is the most preferred between those surveyed (63.3%) (Q18 “How do you manage the sphenoid sinus mucosa”), and 36.4% of them totally rip it. Venous bleeding is usually managed by irrigation with warm saline solution, hemostatic agents, elevation of the head, and avoiding positive expiratory pressures.

### 4.3 Identification of the sellar floor: variable degrees of pneumatization and septation of sphenoid sinus

The sphenoid sinus can present with highly variable degrees of pneumatization and septation. Upon entering the sphenoid sinus, the number, positioning and orientation of sphenoidal septa must be recognized as per CT and/or MRI depiction: they can sometimes delineate “false” chambers inside sphenoid sinus. It is crucial to remove all septa within the sphenoid cavity in order to expose all the bony prominences and depressions at posterior wall of the sphenoid sinus: sellar prominence, medial and lateral optico-carotid recesses, parasellar carotid prominences, and clival recess. The septations eventually attach to internal carotid arteries protuberances, thus fracturing or sheering should be avoided, but removed with Kerrison rongeurs or microdrill. According to Hamid et al. (2008), the sphenoid sinus can be classified as conchal, presellar, sellar, and postsellar. In cases of presellar and conchal pneumatization of sphenoid sinus, there is paucity of landmarks for orientation. The conchal type, which is the rarest, is poorly pneumatized and the area below the sella turcica is a solid block of bone. Presellar sinus is pneumatized until the anterior wall of the sella. In the sellar type (most common), pneumatization reaches the posterior margin of the sella. In the postsellar type, pneumatization extends beyond the sella into the dorsum sellae and posterior clinoids (Wiebracht and Zimmer, 2014). The bony pneumatization of the sphenoid sinus is essential and must be taken into consideration. Indeed, in patients with a presellar or conchal type sinus, bony landmarks for the optic nerves and ICAs are not easily identifiable, and the risk of injury is higher. In these cases, the surgeon should constantly focus to maintain the midline, and choose for image-guided system support.

### 4.4 Sellar floor opening

Once the sellar floor has been identified, the mucosa over the floor of the sella is removed. Boundaries for removal of the sellar floor are the tuberculum sellae or suprasellar notch superiorly, the clival indent inferiorly, and the carotid prominence laterally. In cases of sellar floor erosion by the tumor, the Kerrison punch or curette are used to open it, whereas diamond drill opening is required whether if the sellar floor is intact, starting at the inferior aspect of the floor on the midline. Vast majority of panelists (90.9%) concerning the modality of sellar floor opening (Q19) prefer sellar bone floor removal, whilst minor percentage run a bone flap without bone resection. In case of microadenoma (Q20 “In case of microadenoma how you perform the sellar floor opening?), 54.5% of the interviewees complete a tailored sellar floor opening, whilst others stick upon conventional complete sellar floor opening. Elevating the dura off the floor of the sella in a medial to lateral direction can help in defining its width up to the cavernous sinuses: the dura at this level is densely adherent and will not elevate during this maneuver.

### 4.5 Circular sinuses bleeding management

The superior and inferior intercavernous sinuses should be identified before the dural incisions: they can be more evident, especially in case of a small sella (not enlarged by the tumor), such as in cases of microadenomas; sometimes the entire sella can be covered by a large venous plexus. The opening in the dura is made with a retractable knife in the center, starting inferiorly and then extending superiorly. During the opening, sometimes it is necessary to close the intercavernous sinus to expose the tumor inside the sella. In case of venous bleeding, two tools are available to control further hemorrhage: hemostatic agents and bipolar coagulation. The use of monopolar coagulation due to its thermal dispersion and the placement of metal clips is not recommended. Thrombin-based hemostats are extremely effective in controlling any bleeding and it may be applied several times during the opening of the dura mater. Bipolar coagulation is also effective, however the two meningeal sheets through which the venous sinus passes (dural and periosteal) may not always be clearly visible. Coagulation should not take place too close to the cavernous sinus, because of the higher risk of greater bleeding due to further tearing of the dura leaflets or direct damage to the ICA.

## 5 Tumor removal strategy

### 5.1 Pituitary microadenoma

The sellar dura must be opened extensively to expose the interface between the pituitary gland and the adenoma and allow, where possible, an extracapsular dissection of the adenoma. The initial opening of the dura should not transgress the pituitary gland or adenoma. Angled microdissectors are then used to separate the dura off the underlying tumor and/or gland. The opening in the dura is enlarged in a cruciform manner as needed with the use of a microscissors or round scalpel. The height of diaphragma sellae should be kept in mind when extending the opening of the dura superiorly in order to avoid breaching of the arachnoid of the suprasellar cistern and therefore a CSF leakage.

### 5.2 Adjuvant fluorophore

Differentiation of PitNETs tissue from surrounding normal tissue during surgery can be challenging in some cases. Several fluorescent agents have been proposed for the use in pituitary surgery, with the aim of improving tumor resection rate. Indocyanine green fluorescence was measured 4 times higher in tumor tissue, so that is considered effective in distinguishing it from normal pituitary tissue and neurovascular structures (Chang et al., 2019; Vergeer et al., 2022). 5-Aminolevulinic acid (5-ALA) was investigated was found able to detect microadenomas, even when MRI was negative but conclusive results seem controversial so far (Chang et al., 2019; Eljamel et al., 2009; Nemes et al., 2016; Neumann et al., 2016). Finally, OTL38 can potentially serve as a selective fluorescent agent in non-functioning pituitary adenomas in the near future (Vergeer et al., 2022).

Anyway (Q22 “Do you use intraoperative fluorescence?“), upon the need of adjuvant fluorophore there is a 66.6% rate of responding panelists that uses “Fluorescein” and 33.3% “Indocyanine green” fluorophore during endoscopic endonasal surgery for pituitary adenomas.

### 5.3 Pituitary macroadenoma

**Fig. 5:**
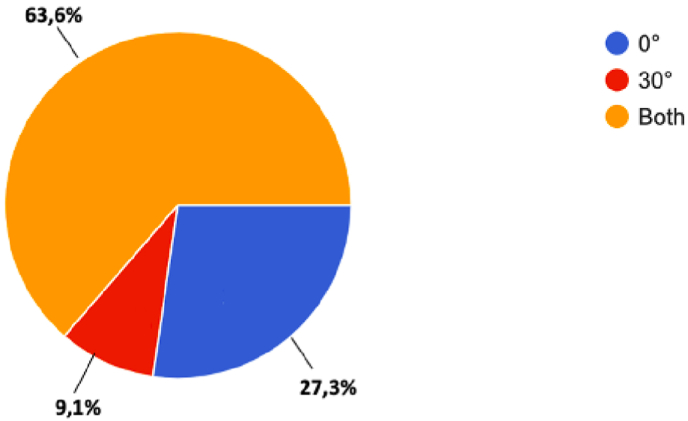
Question 21 “Which type of endoscope do you use for tumor removal?”

The key factors for a successful resection are related to intrinsic tumor features: the anatomical relationships between tumor and critical neurovascular structures, subarachnoid invasion, and tumor consistency (Koutourousiou et al., 2013) (Cappabianca et al., 2014b) (Nishioka et al., 2017; Goel et al., 2004). When the tumor is firm, rubbery or fibrous (e.g., in some cases of recurrent tumor) the possibility of completing safe GTR is lower. A predominantly vertical axis of growth reduces the likelihood of neurovascular structures involvement, whilst any eccentric growth into the lateral aspects of anterior and/or middle cranial fossae hinders the resection via endonasal corridor. Tumor removal starts at the intrasellar inferior aspects of tumors, then continue with the lateral extents, and finally – if any-with the suprasellar component: these maneuvers aim to not having the suprasellar cistern falling into the sella and thus blocking access to posterior components of the mass. The cleavage plane has to be identified first between medial walls of cavernous sinuses and then gradually up to the arachnoid of the diaphragma sellae. This is best achieved by applying traction to the tumor itself using the suction without pulling with grasping forceps to spare the pituitary gland tissue. For tumors with a soft consistency, a two-suction technique is a suitable method of resection. In the case of a firm tumor with a pseudocapsule, extracapsular dissection is performed with identification and preservation of the normal pituitary gland. It should be minded that upon tumor removal, if the diaphragm descends unevenly, there can be a residual tumor left behind: a Valsalva maneuver (up to 30–40 mmHg of intrathotacic pressure) can rule out this suspect and eventually detect any breach of the arachnoid hiding CSF leak. After resection, the cavity can be explored with a 30-degree or 45-degree endoscope and by gentle retraction of the arachnoid folds to ensure the absence of any tumor remnant. (Q21 “Which type of endoscope do you use for tumor removal?“) Most part of the interviewees (63.3%) affirmed to use both 0-degree and 30-degree endoscope during tumor removal phase, 27.3% runs resection with sole 0-degree endoscope, and 9.1% with a 30-degree endoscope [Fig. 5].

### 5.4 Intrasellar venous bleeding management

Venous bleeding typically arises from the epidural space, cavernous and intercavernous sinuses. Higher blood pressure and/or anti-Trendelenburg position can be adequate to reduce epidural bleeding. Massive bleeding tends to be best dealt with by applying topical hemostatic agents, irrigation with warm saline solution or gentle cottonoids compression; on the other side, tumor bleeding is more easily controlled with cottonoid compression or hemostatic agents such as oxidized cellulose and microfibrillar collagen.

### 5.5 Macroadenomas partial resection

Partial debulking like in case of dumbbell shaped adenomas that breach a narrow neck diaphragm has been associated with a higher risk of incomplete resection and thus residual tumor hemorrhage. This results in higher rates of morbidity and mortality, related to mass effect due to abrupt increase of lesion volume, i.e. compression of the optic pathway, acute hydrocephalus, and/or nerve injury or lately damage related to vasospasm. If residual tumor is suspected, a neuroradiological exam (e.g., CT scan) should be performed soon after surgery to rule out post-operative hemorrhage and the risk of vasospasm whose prevention can back up on post-SAH vasospasm management guidelines.

## 6 How to manage ICA

### 6.1 Prevent ICA injury

The major risk of ICA injury occurs in cases with parasellar extension of the PA, or in cases of a narrow paracavernous ICA, and in GH-secreting PAs. The key to prevent this potentially devastating complication is based on thorough analysis of preoperative imaging, the intraoperative use of a Doppler probe, neuronavigation, and detailed knowledge of the anatomy [Fig. 2, Fig. 3]. (Labib et al., 2014)

### 6.2 Intraoperative management of ICA injury

Both surgeons and anesthesia staff need to be actively involved upon this event: Injury to the ICAs is usually evidenced by a brisk bleeding that must be controlled immediately while maintaining adequate cerebral perfusion. Hypotension as an attempt to control the bleeding is contraindicated because it can cause cerebral hypoperfusion and hypoxia. Heparin may be counterintuitive to avoid embolic phenomena, but it is crucial to prevent a stroke (Chin et al., 2016) (Rowan et al., 2018; Kassir et al., 2021). Visualization of the lesion site may be aided by inducing transient hypotension with the use of adenosine, a nucleoside analog used in the surgical procedure for the clipping of intracranial aneurysms (Meling, 2018): during endoscopic endonasal surgery procedures (Fastenberg et al., 2019; Nwosu et al., 2021) it induces a transient and moderate hypotension, which offer better chances of visualization over the ICA lesion sites and thus its repair. This event is best managed by two surgeons in a four-handed technique under dynamic endoscope view. As happens in vascular surgery, two large suction cannulas can help controlling the brisk bleeding and achieve identification of its source at the artery that is later compressed with diligent packing (e.g., muscle or fat with or without fibrin glue); a control of bleeding with the balloon catheters through nasal cavity to occupy the entire nasopharynx and sphenoid cavity has also been reported (Chin et al., 2016).

Upon achievement of resilient hemostasis, the patient is transferred immediately to an angiography suite for endovascular assessment and treatment. Risks of re-bleeding, stroke, cranial nerve deficits, and death remains high in the first 48 hrs, whereas long-term complications include pseudoaneurysm or carotid cavernous fistula (Chin et al., 2016; Cossu et al., 2020).

## 7 Sellar closure

Over the years, multiple materials have been employed to close osteo-dural breach and various techniques have been proposed for intra-dural and/or extradural closure of the sella with or without packing of the sphenoid sinus (Sigler et al., 2017; Citardi et al., 2000; Leng et al., 2008; Cappabianca et al., 2002). Key points for reconstruction as final step of the endoscopic endonasal procedure should be: obliteration of any dead space, isolation of the intradural compartment from the sinonasal tract, promotion of healing processes, and prevention of intracranial pressure raise (limiting strains, such as cough, bending over and so on). Autologous graft material (e.g., fat, fascia, nasal mucosae) is preferred over heterologous materials (dural substitute) since these are promptly available, decrease the risk of infections and, above all, promote tissue regeneration, thus favoring healing process. Overpacking of the sella cavity may result in compression of the optic system and must be avoided. Packing of nasal cavities is usually not necessary, except in cases with copious bleeding from the mucosa, as in patients with uncontrolled hypertension or harboring GH or ACTH secreting lesions (Acromegaly and Cushing).

**Fig. 6:**
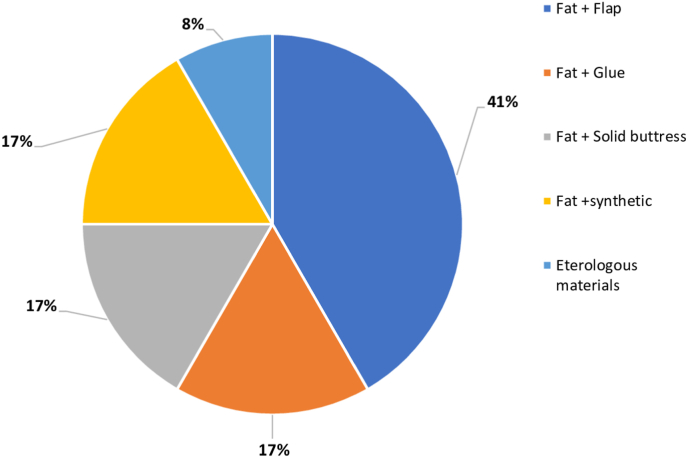
Question 28 “What reconstruction do you use in case of Macroadenoma with CSF leakage”.

Reconstruction techniques are tailored according to the size of osteo-dural defect (Esposito et al., 2007; Conger et al., 2018):

• If CSF leak is not detected, the sella is filled with absorbable hemostatic sponge and a fibrin glue; sphenoid sinus packing is not mandatory. Notwithstanding no evidence of intraoperative CSF leak in case of microadenoma (Q23), 54.5% of interviewees prefer to perform reconstruction; in case of macroadenoma with no leak (Q26), 36.4% prefer to perform sellar reconstruction, being autologous materials (Q27) preferred in most cases (63.6% autologous and 18.2% heterologous)
• When a small leak is seen albeit any breach is evident, it can be useful to seal by mean of bipolar coagulation the arachnoid of the suprasellar cistern at sellar dura mater, the so-called “de Tribolet's point”. However, the 63.6% of delegates respondents prefer to perform reconstruction (Q24 “What reconstruction do you use in case of Microadenoma with CSF leakage”) by mean of both, sellar packing and closure in case of microadenoma with any intraoperative CSF leak with a large prevalence of use for autologous materials 72.7% (Q25).
• In case of large osteo-dural following resection of Macroadenoma (Q28), those who surveyed addressed the need for multilayer intradural-extradural reconstruction. The fat graft is widely adopted among the delegates: it is molded to fit inside the surgical cavity and across the bone dural defect, reaching the extradural space). According to the most part of interviewees the naso-septal flap is useful to bolster the reconstruction, two of them adopt a semisolid buttress to sustain repair materials and only one uses synthetic tissue in the reconstruction [Fig. 6].

To reduce CSF pulsation and intracranial pressure, semi-sitting or sitting position is suggested during recovery: 63.6% and 18.2% respectively prefers to raise the head of 30° and 45° if there no leak is observed (Q29 “Elevation of the trunk in case of no intraop CF leakage”), 72.7% and 27.3% that respectively prefers to raise the head 30° and 45° upon the occurrence of intraoperative CSF leakage (Q30 “Elevation of the trunk in case of intraop CF leakage”); this recommendation is kept for almost 2 weeks after discharge. For the same reasons (Q31 “Mobilization of the patient without any preoperative problem”) 45.5% of surgeons prefers to have the patient out of bed on POD0 and POD1, while in 9.1% prefer mobilization after POD1.

## 8 Postoperative management

### 8.1 Antibiotics

The patient receives two doses of antibiotics as perioperative prophylaxis: the first within 30 minutes before surgery and the second dose 4 hours after. Intravenous cefazolin is usually administered (1st generation cephalosporin at a dose of 1000 mg), whereas patients with a history of allergy, receives intravenous clarithromycin at a dose of 500 mg. In cases of intraoperative CSF leakage, patients should receive a different antibioprophylaxis regimen consisting of a 3rd generation cephalosporin alone or in combination with beta-lactam drug. The use of antibiotics type and duration may vary according to presence of CSF leak: the 63.6% of respondents use Cefazolin and a minor percentage Cefuroxime (18.2%) (Q32 “Which antibiotic do you use without CSF leak?“), all prefer short term protocol (Q33 “duration of antibiotic therapy); the choice is different in case of intraoperative CSF leakage, with a slight prevalence for Cefuroxime (40%) over Cefazolin (30%) (Q34 “Which antibiotic do you use with CSF leak?), with mean preferred duration of 3–5 days (Q35 “duration of antibiotic therapy).

### 8.2 Postoperative endocrinological assessment

The most frequent endocrinological complication during the postoperative course are represented by Diabetes insipidus (DI) and Syndrome of inappropriate antidiuretic hormone secretion (SIADH). DI may lead an electrolyte imbalance and related metabolic complications; therefore, it should be treated in a prompt manner with adequate dosage of desmopressin (1-deamino-8-D-arginine vasopressin, DDAVP). As opposed to DI, SIADH may occur at POD 7–10 in a patient with normal postoperative sodium levels. One of the possible endocrinological complications after pituitary surgery is the syndrome of inappropriate secretion of antidiuretic hormone (SIADH), ranging from 1.8% to 37%, a disorder caused by overproduction of ADH (Kristof et al., 2009). Hyponatremia should be suspect in case of nausea, dizziness, confusion and seizures. Differential diagnosis includes the cerebral salt wasting, characterized by hyponatremia with elevated urine sodium and hypovolemia; in this case an intravenous administration of isotonic or hypertonic fluids to obtain positive fluid balance and correct volume depletion. Hyponatremia from SIADH is usually treated with fluid restriction and/or hypertonic saline, depending on serum Na levels, taking care to not rush the correction. Some authors demonstrate the role of tolvaptans (arginine-vasopressin receptor antagonists) for the treatment of hypervolemic and euvolemic hyponatremia. Indeed, tolvaptan administration proved more effective than fluid restriction treatment (with or without hypertonic saline), with significant higher 24- and 48-h sodium correction rates (Indirli et al., 2021). A 1.0 L water has been recommended by Laws’ group at discharge, and patients are instructed to limit fluid intake per day (Burke et al., 2018). On discharge, special attention is needed to monitor sodium levels in the next 2 weeks postoperatively as outpatient.

Other possible complications include hypocortisolemia and panhypopituitarism. In case of preoperative hypocortisolemia, during the peri-operative period the patient receive replacement with hydrocortisone. Although no defect in detected in any axis, hydrocortisone is administered from POD1 up to endocrinology evaluation within 15/20 days. Correction of central adrenal insufficiency and diabetes insipidus is essential before surgery, if needed, levothyroxine should also be started for treatment of hypothyroidism before the correction of hypocortisolism.

### 8.3 Nasal rinsing

Common postoperative nasal discomforts include nasal crusting, discharge, and obstruction. These problems are caused by the alteration of nasal airways structures and sinus scar tissues: nasal crusting during the immediate postoperative period is usually best managed with saline nasal irrigation. Transient hyposmia may be due to crusting or inflammation of the nasal mucosa. With mucosa-sparing surgery and careful postoperative care (topical nasal spray), functional sinonasal recovery and quality of life are restored within 3 weeks. Interviewees suggest nasal rinsing at 10 or 15 days after surgery (Q36 “Postoperative nasal rinsing: how far from surgery), each apart in 27.3% of cases and is performed usually for (Q37 “Postoperative nasal rinsing: for how long?“) 15 days (18.2% of responders) and 30 days (36.4% of the interviewees). Along 4–6 weeks patients need avoiding straining and any action that favor Valsalva maneuvers (e.g., constipation) and/or leaning the head forward.

### 8.4 Nasal endoscopy

An endoscopic exploration of the nasal cavity (Q38 “Nasal cavity post-op endoscopic control, in which case”) is run upon the occurrence of CSF leak, epistaxis, extensive nasal crusting or bad smell.

### 8.5 Postoperative CSF leakage management

**Fig. 7:**
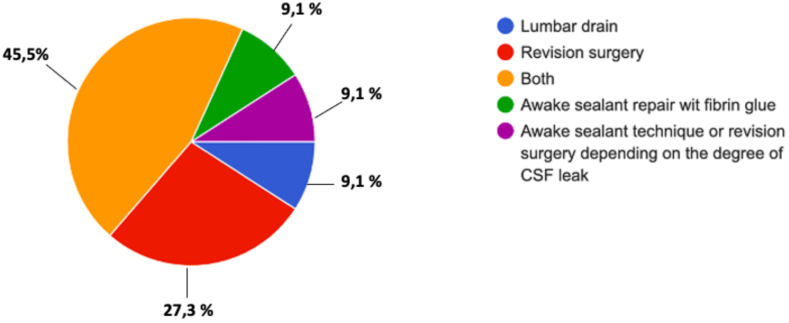
Question 39 “In case of CSF what do you do?”

Interestingly upon the occurrence of postoperative CSF leakage (Q39 “In case of CSF what do you do?“), 45.5% of respondents prefer to attempt revision surgery along lumbar drain placement, 27.3% a revision surgery only, 9.1% an awake sealant fibrin glue injection, and 9.1% a lumbar drain placement. In 9.1%, awake sealant technique or revision depends on the degree of CSF leak [Fig. 7]. Repeated awake sealant fibrin glue injection is usually adequate to arrest small CSF leak (Cavallo et al., 2014).

### 8.6 Antithrombotic prophylaxis

Deep vein thrombosis (DVT) as a complication of endoscopic skull base surgery is directly linked to bed rest and rarely can be caused to surgical procedure itself (when extensive bone drilling is performed). The day after surgery prophylactic anticoagulation is administered at least for seven days (Enoxaparine). Several other precautions such as controlled pressure stockings, compression devices may be considered to reduce the risk of DVT, in case of prolonged bed rest or in patients with known thrombotic risk factors (Q4o “DVT prevention: when and how long”), such as those with a prior history of DVT, Cushing disease, and in these cases antithrombotic prophylaxis is advised for a longer period (up to 3 months) (Rabiei et al., 2023).

### 8.7 Postoperative control

**Fig. 8:**
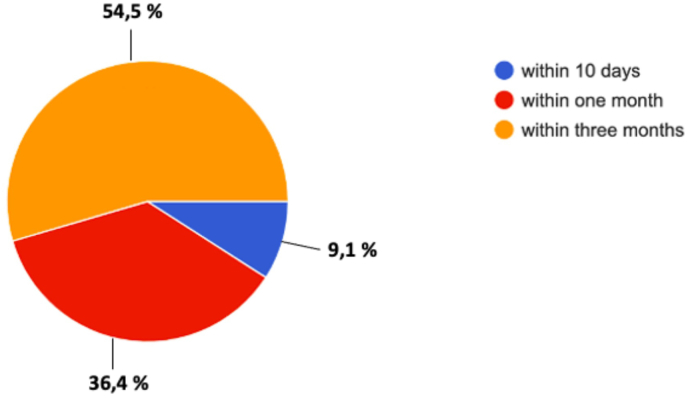
Question 42 “First outpatient clinic access after discharge”.

Concerning the postoperative imaging (Q41 “In-Hospital postoperative imaging?“), 45.5% of the panelists responding, do not perform neuroradiological exam, 36.4% prefer to perform MRi within 48 hours, and 18.2% an early CT scan. All request a Contrast-enhanced MRI (Q43 “Follow-up postoperative imaging”) at 3 months to plan any further treatment. Clinical assessment with formal visual field examination and full endocrinological assessment is advocated at 1 month; concerning the neurosurgical outpatient access for 54.5% of the respondents (Q42 “First outpatient clinic access after discharge”), first outpatient access is fixed at 3months, for 36.4% at 1 month, and for 9.1% within 10 days [Fig. 8].

## Conflict of interest

The authors report no conflict of interest concerning the materials or methods used in this study or the findings specified in this paper.
